# Cataract Surgery during the COVID-19 Pandemic: Insights from a Greek Tertiary Hospital

**DOI:** 10.3390/geriatrics7040077

**Published:** 2022-07-26

**Authors:** Sevasti Tsironi, Dimitrios Kavvadas, Georgios Delis, Alexandra Bekiaridou, Viktoria Kapourani, Fragkeskos Loizou, Panagiota-Sofia Apostolidou, Konstantina Misiou, Efstratios Theofrastou, Thaleia Panakleridou, Eleni Psimenidou, Anastasia Sarafi, Elie Fadel, Sofia Karachrysafi

**Affiliations:** 1 Department of Ophthalmology, General Hospital G. Papanikolaou, 57010 Thessaloniki, Greece; stsironi@yahoo.gr (S.T.); v.kapourani@gmail.com (V.K.); frangeskosl@gmail.com (F.L.); panapostolidou@yahoo.gr (P.-S.A.); kmisiou1992@gmail.com (K.M.); stratos.theofrastou@gmail.com (E.T.); thaleiapanak@gmail.com (T.P.); psim_el@hotmail.com (E.P.); sarafinastasia@gmail.com (A.S.); fadelelie@yahoo.gr (E.F.); 2 Laboratory of Histology-Embryology, Faculty of Health Sciences, School of Medicine, Aristotle University of Thessaloniki, 54124 Thessaloniki, Greece; ampekiaridou@gmail.com; 3 Laboratory of Pharmacology, Faculty of Health Sciences, School of Veterinary Medicine, Aristotle University of Thessaloniki, 54124 Thessaloniki, Greece; delis@vet.auth.gr

**Keywords:** cataract, COVID-19, surgery

## Abstract

Background: COVID-19 has affected everyday clinical practice, having an impact on the quality of healthcare provided, even in eye clinic departments. The aim of this study is to evaluate the consequences of this worldwide pandemic on cataract surgery in a Greek tertiary university hospital. Methods: A total of 805 patients were included in this study. The number of cataract surgeries (CS), the type, the unilateral or bilateral appearance as well as the stage of cataract were recorded for the months between January and June 2019 (pre-COVID period) and compared with the same period in 2021 (during the pandemic outbreak) in the Department of Ophthalmology of Thessaloniki General Hospital G. Papanikolaou. Results: A significant reduction in the number of CS as well as a significant increase in advanced and/or bilateral cataracts in 2021 compared to the pre-COVID period were observed. Conclusions: The COVID-19 pandemic has affected equally the value of ophthalmic interventions as well as the patients’ quality of life, being a powerful reminder of the significant physical and psychological benefits of CS, especially for older adults and patients with comorbidities.

## 1. Introduction

Since the first outbreak of the new coronavirus disease-19 (COVID-19) in Wuhan, China, in December 2019, the virus has affected the lives of billions of people worldwide, having an enormous socioeconomic impact. The COVID-19 pandemic has altered the organization of the health care system, directly impacting the management of surgical patients [1]. As a result of this situation, ophthalmological patients have been affected deeply by an increase in the waiting times and the limited access to cataract surgery (CS) in many healthcare settings (especially in public-funded healthcare systems) due to the prolonged cessation of elective cataract surgery [2].

In fact, some European countries recorded a 97% reduction in CS volume between March and April 2020 compared with the same period in 2019 [3]. The following progressive deterioration of the visual function in patients on waiting lists has a negative impact on their quality of life and psychological state, especially in the elderly population. This study aims to evaluate the effects of COVID-19 on CS patients, comparing the pre-COVID-19 period with the pandemic outbreak.

## 2. Materials and Methods

This is a retrospective, observational cohort study. The electronic medical records of the patients included in this survey were collected from the Department of Ophthalmology of General Hospital G. Papanikolaou, a tertiary hospital in Thessaloniki, Greece. Patients’ records were retrospectively assessed regarding their age, sex, type of cataract subdivided by (a) immature—subcapsular, nuclear, and cortical, (b) and mature—white cataract, and localization of cataract (unilateral or bilateral). Written informed consent was obtained from the patients for anonymized patient information to be published in this article. The study was conducted in compliance with the Declaration of Helsinki.

To fulfill the aims of this study, we compared two equally-spread periods: (i) January to June 2019 (pre-COVID-19 period) and (ii) January to June 2021 (COVID-19 outbreak period). The period in question did not involve any change in rationing to cataract surgery when compared to the prepandemic period being studied.

### Statistical Analysis

Contingency tables were constructed and analyzed using the Pearson chi-square or Fisher’s exact tests for frequencies of nominal. A *p* value of less than 0.05 was considered statistically significant. Data management and statistical analysis were performed using IBM^®^ SPSS^®^ Statistics software v.25 (Armonk, NY, USA).

## 3. Results

**Figure 1:**
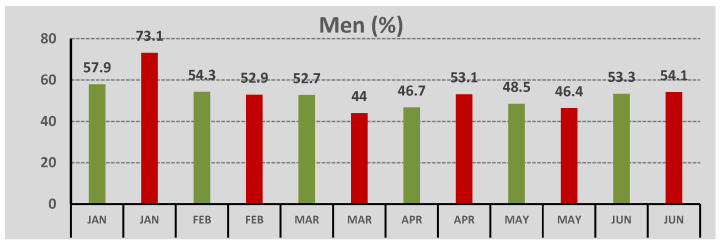
Percentage of men among patients who underwent CS across different months of the pre-COVID-19 (in green color) and the COVID-19 period (in red color).

The main findings were: (i) a significant reduction in the number of CS, (ii) an increase in white mature cataract cases during the pandemic. The total number of CS in the pre-COVID-19 period was 557, and during COVID-19 pandemic, it was 248. Regarding sex, in the pre-COVID-19 period, the percentage of men was 52.1%, and in the COVID-19 period, it was was 52.8%, as demonstrated in Figure 1, without statistical difference in sexes between the two periods (*p* = 0.903).

**Figure 2:**
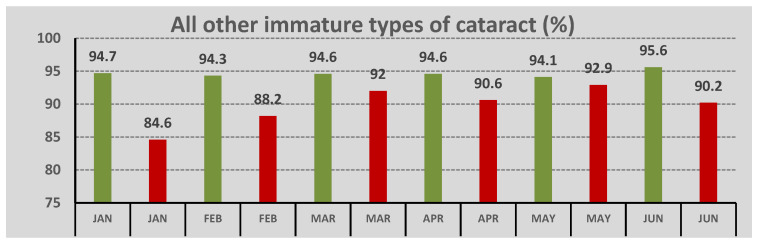
Distribution of immature types of cataract across different months of the pre-COVID-19 (in green) and the COVID-19 period (in red).

Concerning the type of cataract in the pre-COVID-19 period, 5.4% of the cases were of the white type, whereas 94.6% of the cases included all other immature types of cataracts (Figure 2).

**Figure 3:**
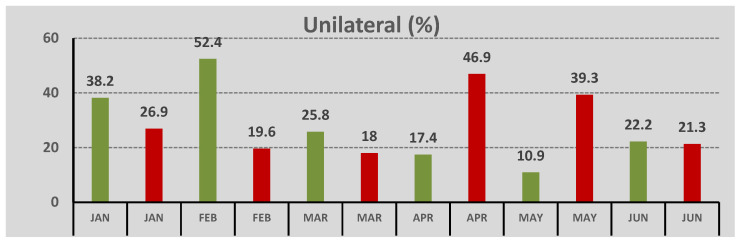
Localization of cataract (unilateral or bilateral) across different months of the pre-COVID-19 (in green) and the COVID-19 period (in red).

In COVID-19 period, the immature types constituted 89.9% of all types, and the white type was 10.1%. The difference in the prevalence of the white cataract between the two periods was statistically significant (*p* = 0.022). In the pre-COVID-19 period, 27.8% of the submitted cases concerned unilateral cataracts, whereas 72.2% of the cases represented bilateral cataract (Figure 3). During the pandemic outbreak, 26.2% of the cataracts were unilateral, and 73.8% were bilateral with no statistical significance between the two periods (*p* = 0.697). Nevertheless, in the pre-COVID-19 period, when the rates were compared monthly, there was a higher rate of unilateral cataract in January and February and a higher rate of bilateral cataract in April and May (*p* < 0.001). In the COVID-19 period, there was a higher percentage of unilateral cataract in April (*p* = 0.022).

## 4. Discussion

Undoubtedly, the COVID-19 pandemic has forced massive changes in everyday clinical practice. This was also assessed by the reduction in cataract (non-urgent) surgeries (CS) performed at least in Thessaloniki General Hospital G. Papanikolaou. CS is one of the most performed procedures, and it has proven to be one of the most cost-effective healthcare interventions [3]. This surgery has a significant impact on the patient’s everyday life and wellbeing, and its postponement can lead to the aggravation of the patient’s clinical presentation. Patients’ insecurity and fear were depicted in the increase in missed ophthalmology clinic visits worldwide [4].

The patient’s safety and the safety of healthcare providers is a prerequisite for the performance of a clinical procedure, especially during any pandemic. The ocular examination necessitates face-to-face contact with patients, and the danger of viral shedding from the respiratory system might be a significant concern for ophthalmologists [5]. With the onset of the COVID-19 pandemic, many aspects of the SARS-CoV-2 were unknown, including its virulence and transmissibility. Research on COVID-19 is fast progressing, and multiple hypotheses about various pathways of transmissibility have been proposed [6]. Whether the eyes are a potential transmission source remains obscure, but the evidence so far supports that ocular transmission is possible [6]. Thus, correct prevention strategies are essential for healthcare providers and patients. Regarding ocular manifestations of COVID-19, the main symptom reported is conjunctivitis [7]. An antiviral ocular treatment to reduce the viral load on the conjunctiva of patients and limit the virus ocular transmission could be useful [7].

The increase in white cataract cases suggests the negative impact of delayed patient management. In the COVID-19 period, there was a decrease in CS, but a significant increase in the white–mature type of cataract. Clinically, this depicts that the postponement of the CS due to the COVID-19 pandemic led to cataract progression to the mature and hypermature type. Therefore, the increase in the incidence of the mature white cataracts during the pandemic could be explained based on two major factors; (i) due to the increase in the waiting time of patients on the lists for cataract surgeries, (ii) the prolonged postponement of visitations to the ophthalmologists due to the patients’ fear of infection by COVID-19.

Patient management during the pandemic brought up new ways of everyday clinical practice, such as telemedicine and one-day cataract surgery [2,8]. Firstly, there were reports of pediatric CS practices that used telemedicine to arrange appointments or followup with patients who underwent CS. Moreover, practices such as the bilateral same-day CS emerged to minimize the healthcare-related exposures for patients [9].

### Strengths and Limitations

This study includes a “real-world” cohort of patients, thus depicting the real problems in everyday clinical practice. Nevertheless, it is a retrospective study from one Greek department, allowing the investigator to choose the time periods for the study, inviting selection bias.

## 5. Conclusions

We designed a retrospective observational study to review the impact of the COVID-19 pandemic on the management of cataract patients. We decided to proceed with a retrospective study as it would be more appropriate and accurate to gather the data between corresponding time periods before and during the pandemic. Further data collection and a prospective study could be an extension of the present research project. The COVID-19 pandemic has caused a significant reduction in the number of CS performed. A significant increase was recorded in the submitted cases of white mature cataracts, proving the pandemic’s negative impact on patients’ management, including the delayed surgical therapy. The COVID-19 pandemic has equally affected the value of ophthalmic interventions and the patients’ quality of life, being a powerful reminder of the significant physical and psychological benefits of CS, especially for older adults.

## Data Availability

Not applicable.
